# Genetic and structural characterization of 20 autosomal short tandem repeats in the Chinese Qinghai Han population and its genetic relationships and interpopulation differentiations with other reference populations

**DOI:** 10.1080/20961790.2018.1485199

**Published:** 2018-07-18

**Authors:** Zhanhai Wang, Bin Lu, Xiaoye Jin, Jiangwei Yan, Haotian Meng, Bofeng Zhu

**Affiliations:** a The Public Security Bureau of Qinghai Province, Xining, China;; b Key Laboratory of Shaanxi Province for Craniofacial Precision Medicine Research, College of Stomatology, Xi’an Jiaotong University, Xi’an, China;; c Clinical Research Center of Shaanxi Province for Dental and Maxillofacial Diseases, College of Stomatology, Xi’an Jiaotong University, Xi’an, China;; d Research Center of Stomatology, Stomatological Hospital, Xi'an Jiaotong University, Xi’an, China;; e Beijing Institute of Genomics, Chinese Academy of Sciences, Beijing, China

**Keywords:** Genetic polymorphisms, forensic genetics, phylogenetic reconstruction, short tandem repeat

## Abstract

China is a multinational country composed of 56 ethnic groups of which the Han Chinese accounts for 91.60%. Qinghai Province is located in the northeastern part of the Qinghai–Tibet Plateau, has an area of 72.12 km^2^, and is the fourth largest province in China. In the present study, we investigated the genetic polymorphisms of 20 short tandem repeat (STR) loci in a Qinghai Han population, as well as its genetic relationships with other populations. A total of 273 alleles were identified in 2 000 individuals at 20 loci, and the allelic frequency ranged from 0.000 2 to 0.532 7. The 20 STR loci showed a relatively high polymorphic rate in the studied group. Observed and expected heterozygosities ranged 0.613 0–0.907 5 and 0.614 8–0.920 0, respectively. The combined power of discrimination, and the probability of exclusion in duo and trio cases were 0.999 999 999 999 999 999 999 999 34, 0.999 996 0 and 0.999 999 996 5, respectively. Analyses of interpopulation differentiation revealed that the most significant differences were found between the Qinghai Han and Malaysian, while no significant differences were found between the Qinghai Han and Han people from Shaanxi and Jiangsu. The results of principal component analysis, multidimensional scaling analysis and phylogenetic reconstructions also suggested the close relationships between the Qinghai Han and other two Han populations. The present results, therefore, indicated that these 20 STR loci could be used for paternity testing and individual identification in forensic applications, and may also provide information for the studies of genetic relationships between Qinghai Han and other groups.

## Instruction

The Han population is the largest of the 56 officially recognized ethnic groups in China. Findings from the 6th National Population Census of 2010 suggest that they make up 91.60% of the overall Chinese population with a population of 1 220 844 520, and they are also distributed worldwide. The Chinese language, used as the spoken and written language of Han people, belongs to the Sino–Tibetan language family. The appellation ‘Han’ can be traced back to the Han dynasty of the second and third centuries and represents the majority of the Chinese population to date.

Qinghai Province has the fourth largest land area in China. It is located in the northeastern part of the Qinghai–Tibet Plateau, which has an altitude over 3 000 m above sea level. The history of Qinghai Province began during the Han dynasty when General Huo Qubing built the military fortress known as Xipingting. This was the former site of Xining, which appeared during the Ming dynasty between 1368 and 1644. In 2010, Qinghai had a population of 5 626 723, of which 39% was taken up by minorities including Tibetans, Mongolians, Kazaks and the Hui, Tu and Salar.

In the present study, the genetic distributions of 20 short tandem repeat (STR) loci and the sex-determining locus amelogenin were studied in the Qinghai Han population. Additionally, the phylogenetic relationships between the Qinghai Han and other reference populations [1–13] were studied using 13 overlapping STR loci including *D8S1179*, *D21S11*, *D18S51*, *vWA*, *D3S1358*, *FGA*, *TH01*, *D5S818*, *D13S317*, *D7S820*, *CSF1PO*, *D16S539* and *TPOX* loci.

## Materials and methods

### Sample collection and DNA extraction

Blood samples were collected from 2  000 unrelated healthy Han individuals living in Qinghai Province, China, whose ancestors over the past three generations were Han individuals who had not migrated or interbred with other ethnic groups. All of the participants signed an informed consent form and completed a questionnaire providing information about their direct blood relatives over three generations. The experimental procedures conformed to the human and ethical research principles of Xi’an Jiaotong University Health Science Center, China. Genomic DNA extraction was performed using the Chelex-100 procedure as described by Walsh et al. [14].

### Polymerase chain reaction (PCR) amplification and STR typing

PCR amplification was performed using the PowerPlex^®^ 21 System (Promega, Madison, WI, USA). The total volume of PCR reactions was 25 µL, containing 5 µL PowerPlex^®^ 21 5× Master Mix, 5 µL PowerPlex^®^ 21 5× Primer Pair Mix, 1 ng template DNA and amplification grade water. Amplification was carried out using a GeneAmp PCR System 9700 Thermal Cycler (Applied Biosystems, Foster City, CA, USA) under the manufacturer’s specifications. The AB PRISM 3130 Genetic Analyzer (Applied Biosystems) was used to obtain sample genotypes. Raw data were analysed using GeneMapper ID 3.2 software (Applied Biosystems). 9947A DNA was used as a positive control.

### Statistical analysis

Allelic frequencies of 20 STRs, their forensic relevant parameters, and *P*-values for exact tests of Hardy–Weinberg equilibrium were calculated using the modified Powerstat (version 1.2) spreadsheet. Linkage disequilibrium (LD) analysis of pairwise STR loci was calculated using Genepop version 4.0.10 [15]. Based on genetic data of the 13 overlapping STRs, genetic differentiation comparisons (*P*-values) between the Qinghai Han population and other referenced populations were conducted using Arlequin software version 3.1 with the method of analysis of molecular variance (AMOVA) [16]. Population genetic structure analysis among the Qinghai Han population and other populations was performed using Structure software version 2.2 [17]. The pairwise genetic distance (*D_A_*) and fixation index (*F*_ST_) of the studied Han group and other populations were calculated using the DISPAN program [18] and Arlequin software version 3.1, respectively. Heatmaps of *D_A_* and *F*_ST_ between these populations were plotted by *R* software (https://www.r-project.org/). Principal components analysis (PCA) of these populations was drawn using MVSP software version 3.1 [19] based on the allelic frequencies of the 13 overlapping STRs. Multidimensional scaling (MDS) analysis of the Qinghai Han population and other compared populations was plotted using IBM SPSS version 18.0 (IBM Co., Armonk, NY, USA). Two different phylogenetic trees were constructed by MEGA software version 5.0 [20] and PHYLIP software version 3.6 to determine the phylogenetic relationships between Qinghai Han and other populations.

## Results and discussion

### Allelic distributions and forensic parameter analysis of 20 STR loci

The allelic frequencies of 20 autosomal STR loci and their corresponding forensic relevant parameters are shown in Table 1. A total of 273 alleles were found in the studied Han population within the 20 loci (Table 1). The minimum allelic frequency was 0.000 2 and the maximum was 0.532 7. The lowest values of the power of discrimination (*DP*), and the probability of exclusion (*PE*) in duo and trio cases were 0.792 6, 0.203 5 and 0.306 8, respectively, at the *TPOX* locus, while the highest values were 0.987 5, 0.720 5 and 0.810 8, respectively, at the *Penta E* locus. With the exception of locus *TPOX*, the polymorphism information content (*PIC*) of all remaining loci reached above 0.6. Highest observed heterozygosity (*Ho*) and expected heterozygosity (*He*) values were observed at the *Penta E* locus, while the lowest *Ho* and *He* values were at the *TPOX* locus. The combined *DP*, and *PE* in duo and trio cases were 0.999 999 999 999 999 999 999 999 34, 0.999 996 0 and 0.999 999 996 5, respectively. These results revealed that these 20 STR loci are highly polymorphic, and have the potential to be used in both forensic human identification and paternity testing in the Qinghai Han population.

**Table 1.** The allelic frequencies and forensic statistical parameters of the 20 STR loci in Chinese Qinghai Han population (*n* = 2 000).

Allele	*D8S1179*	*D21S11*	*D18S51*	*vWA*	*D3S1358*	*FGA*	*TH01*	*D5S818*	*D13S317*	*D7S820*	*CSF1PO*	*D16S539*	*TPOX*	*D2S1338*	*D19S433*	*Penta D*	*Penta E*	*D6S1043*	*D1S80*	*D12S391*
5																	0.053 2			
6							0.103 5	0.000 2								0.005 0	0.000 2			
7							0.255 2	0.014 0	0.001 2	0.001 2	0.002 2		0.000 2			0.007 2	0.002 2			
8	0.000 8						0.059 8	0.003 5	0.266 2	0.145 5	0.002 8	0.009 0	0.532 7			0.042 5	0.006 0	0.000 8		
9	0.000 2		0.000 2				0.519 2	0.068 0	0.138 3	0.063 0	0.053 2	0.266 5	0.122 2		0.000 2	0.313 2	0.008 2	0.002 0		
9.1										0.002 8										
9.3							0.039 0													
10	0.103 2		0.001 8				0.022 8	0.193 0	0.147 5	0.163 5	0.230 5	0.119 0	0.021 5		0.000 2	0.114 0	0.042 2	0.027 8		
10.1										0.000 8										
11	0.072 5		0.001 8				0.000 5	0.337 8	0.241 5	0.339 0	0.242 2	0.258 8	0.291 8		0.003 0	0.160 0	0.134 8	0.098 5	0.062 0	
11.1										0.000 2										
11.2															0.000 8					
11.3												0.000 2								
12	0.125 8		0.029 2		0.001 2			0.240 8	0.159 5	0.238 0	0.383 0	0.214 8	0.028 8		0.040 0	0.181 0	0.106 8	0.130 0	0.039 8	
12.2															0.004 8					
12.3																		0.000 2		
13	0.235 5		0.200 5	0.002 8	0.001 0	0.000 2		0.131 5	0.035 7	0.041 5	0.074 0	0.115 8	0.001 8		0.287 2	0.124 2	0.050 3	0.136 2	0.100 8	
13.2															0.049 2					
14	0.202 0		0.219 2	0.252 5	0.044 2			0.009 8	0.009 5	0.004 2	0.010 7	0.014 5	0.000 8		0.244 2	0.042 0	0.083 0	0.138 0	0.079 2	
14.2															0.112 2					
15	0.172 5		0.178 5	0.033 0	0.373 8			0.001 5	0.000 5	0.000 2	0.001 2	0.001 5	0.000 2		0.067 0	0.009 0	0.095 5	0.016 2	0.302 5	0.016 0
15.2															0.144 8					
15.3																			0.000 8	
16	0.073 0		0.123 5	0.185 5	0.331 5									0.007 8	0.012 2	0.001 8	0.077 5	0.002 5	0.227 2	0.008 5
16.2															0.029 0					
16.3																			0.009 0	0.000 2
16.4																	0.000 2			
17	0.011 5		0.070 3	0.243 2	0.184 0	0.000 5								0.064 8	0.001 8		0.088 8	0.043 5	0.085 5	0.093 8
17.2															0.002 8					
17.3																			0.055 2	
18	0.003 0		0.043 0	0.184 2	0.056 2	0.018 5								0.107 8	0.000 5		0.079 0	0.177 8	0.011 5	0.229 8
18.2																		0.000 2		0.000 2
18.3																			0.023 0	0.001 0
18.4																	0.000 8			
19			0.040 2	0.080 8	0.007 2	0.047 2								0.159 0			0.059 5	0.158 2	0.001 0	0.224 5
19.2																				0.000 2
19.3																			0.002 5	
19.4																	0.000 8			
20			0.036 5	0.017 5	0.000 8	0.054 5								0.127 0			0.044 5	0.052 8		0.164 5
20.2						0.000 5														
20.3																		0.000 5		
21			0.028 0	0.000 5		0.103 8								0.027 2			0.030 5	0.010 7		0.112 8
21.2						0.003 2														
21.3																		0.002 8		
22			0.013 8			0.166 8								0.044 0			0.021 5	0.000 5		0.078 0
22.2						0.006 2														
22.3						0.000 2												0.000 5		
23			0.004 0			0.217 5								0.219 5			0.008 8	0.000 2		0.044 0
23.2						0.009 5														
24			0.005 5			0.188 0								0.159 5			0.004 8			0.016 5
24.2						0.005 2														
25			0.003 0			0.110 8								0.063 0			0.001 0			0.007 5
25.2						0.004 8														
26		0.000 2	0.001 0			0.046 8								0.016 5						0.002 2
26.2						0.001 2														
27		0.002 8				0.010 2								0.003 5						0.000 2
27.2						0.001 0														
28		0.043 0				0.002 8								0.000 5						
28.2		0.009 5																		
29		0.249 8				0.000 2														
29.2		0.003 2																		
29.3		0.000 2																		
30		0.296 0				0.000 2														
30.2		0.015 0																		
30.3		0.005 2																		
31		0.100 8																		
31.2		0.071 8																		
32		0.029 8																		
32.2		0.126 5																		
33		0.004 5																		
33.2		0.037 2																		
34.2		0.003 5																		
35		0.000 2																		
35.2		0.000 5																		
36		0.000 2																		
Ho	0.848 0	0.817 0	0.859 0	0.810 5	0.712 0	0.860 0	0.649 5	0.772 0	0.815 0	0.771 0	0.754 5	0.797 0	0.613 0	0.872 0	0.806 5	0.801 0	0.907 5	0.864 5	0.821 5	0.830 0
He	0.836 8	0.814 2	0.853 0	0.800 8	0.711 4	0.858 5	0.648 9	0.768 5	0.803 1	0.774 8	0.733 1	0.788 1	0.614 8	0.862 2	0.814 7	0.811 3	0.920 0	0.873 3	0.823 9	0.839 6
MP	0.049 2	0.058 3	0.038 8	0.071 0	0.135 4	0.035 2	0.169 4	0.089 9	0.069 1	0.084 0	0.119 5	0.079 8	0.207 4	0.034 6	0.058 0	0.058 6	0.012 5	0.029 7	0.051 0	0.044 9
DP	0.950 8	0.941 7	0.961 2	0.929 0	0.864 6	0.964 8	0.830 6	0.910 1	0.930 9	0.916 0	0.880 5	0.920 2	0.792 6	0.965 4	0.942 0	0.941 4	0.987 5	0.970 3	0.949 0	0.955 1
PIC	0.816 2	0.791 6	0.836 6	0.771 1	0.660 8	0.843 0	0.602 7	0.733 0	0.774 3	0.741 7	0.689 6	0.755 2	0.554 5	0.847 2	0.791 4	0.787 5	0.914 4	0.860 0	0.804 1	0.820 2
PE(D)	0.503 4	0.470 3	0.546 6	0.427 5	0.294 4	0.558 0	0.243 0	0.376 0	0.431 9	0.388 7	0.324 1	0.403 5	0.203 5	0.564 9	0.467 5	0.458 5	0.720 5	0.589 7	0.492 0	0.513 6
PE(T)	0.690 9	0.631 0	0.712 7	0.618 7	0.447 1	0.714 7	0.354 5	0.548 1	0.627 2	0.546 4	0.517 5	0.593 5	0.306 8	0.738 7	0.611 2	0.600 9	0.810 8	0.723 6	0.639 5	0.655 9
*P*	0.000 5	0.879 0	0.528 0	0.616 5	0.925 7	0.756 0	0.598 2	0.400 8	0.622 0	0.701 1	0.040 9	0.615 2	0.913 3	0.383 4	0.816 4	0.645 4	0.007 9	0.127 0	0.064 2	0.479 7

Ho: observed heterozygosity; He: expected heterozygosity; MP: matching probability; DP: power of discrimination; PIC: polymorphism information content; PE(D): probability of exclusion in duo cases; PE(T): probability of exclusion in trio cases; *P*: probability values of exact tests for Hardy-Weinberg equilibrium.

### LD analysis

The results of LD tests are shown in Table 2. After Bonferroni correction, the exact *P*-values of two (*D19S433* and *FGA*; *TH01* and *FGA*) out of the 190 pairwise comparisons were below the significant level (0.000 263). LD can be influenced by many factors, such as selection, the rate of recombination, the mutation rate, genetic drift, the system of mating, population structure and genetic linkage. As the loci are located on different chromosomes, genetic linkage cannot explain the observed LD. However, additional studies are required to determine the role of other factors in LD.

**Table 2.** The *P*-values of linkage disequilibrium of all pairwise STR loci in Chinese Qinghai Han population.

Loci	*D12S391*	*D1S80*	*D6S1043*	*Penta E*	*Penta D*	*D19S433*	*D2S1338*	*TPOX*	*D16S539*	*CSF1PO*	*D7S820*	*D13S317*	*D5S818*	*TH01*	*FGA*	*D3S1358*	*vWA*	*D18S51*	*D21S11*
D1S80	0.155 1																		
D6S1043	0.277 1	0.798 2																	
Penta E	0.694 5	0.815 2	0.280 2																
Penta D	0.025 4	0.705 5	0.006 6	0.419 4															
D19S433	0.080 2	0.277 1	0.061 5	0.261 2	0.304 8														
D2S1338	0.442 3	0.285 2	0.650 1	0.255 3	0.311 5	0.079 7													
TPOX	0.311 2	0.797 6	0.922 3	0.756 6	0.026 3	0.754 2	0.829 4												
D16S539	0.473 8	0.448 4	0.146 5	0.018 7	0.827 8	0.928 1	0.963 5	0.141 0											
CSF1PO	0.000 5	0.403 7	0.017 0	0.531 3	0.470 7	0.321 5	0.931 5	0.763 0	0.153 9										
D7S820	0.800 8	0.056 4	0.578 8	0.000 7	0.950 7	0.252 8	0.262 5	0.899 5	0.126 6	0.311 5									
D13S317	0.182 9	0.283 7	0.998 8	0.025 8	0.193 4	0.338 2	0.193 3	0.239 0	0.404 5	0.202 1	0.475 4								
D5S818	0.325 3	0.367 5	0.142 8	0.805 3	0.207 4	0.382 8	0.718 8	0.904 8	0.732 9	0.473 7	0.083 0	0.697 4							
TH01	0.039 4	0.014 2	0.170 0	0.101 0	0.847 0	0.010 7	0.823 4	0.220 2	0.483 6	0.619 2	0.389 7	0.710 8	0.415 9						
FGA	0.083 6	0.024 4	0.256 4	0.491 6	0.642 0	0.000 0	0.845 8	0.632 9	0.320 0	0.459 2	0.013 9	0.404 0	0.082 9	0.000 0					
D3S1358	0.483 7	0.671 0	0.356 5	0.264 1	0.465 8	0.031 5	0.471 1	0.395 1	0.905 7	0.355 7	0.796 4	0.313 4	0.485 2	0.210 7	0.297 1				
vWA	0.103 7	0.013 6	0.021 4	0.092 9	0.255 2	0.543 6	0.227 6	0.566 6	0.806 2	0.076 0	0.161 5	0.074 6	0.412 4	0.757 2	0.307 7	0.708 3			
D18S51	0.965 6	0.296 1	0.155 6	0.749 8	0.078 5	0.657 0	0.712 3	0.237 0	0.653 5	0.427 5	0.385 0	0.238 0	0.381 6	0.149 6	0.339 9	0.478 6	0.012 4		
D21S11	0.893 1	0.401 4	0.045 7	0.050 6	0.078 1	0.575 3	0.931 1	0.789 7	0.429 5	0.185 6	0.298 2	0.297 4	0.183 1	0.015 1	0.825 5	0.620 9	0.736 6	0.003 7	
D8S1179	0.854 9	0.174 4	0.306 6	0.132 3	0.223 1	0.258 2	0.000 7	0.351 4	0.205 2	0.367 0	0.016 1	0.739 3	0.691 2	0.787 5	0.863 1	0.043 7	0.613 4	0.232 5	0.785 0

### Interpopulation differentiations between the Qinghai Han population and other compared populations

The *P*-values of genetic differentiation comparisons are shown in Table 3. Significant differences (*P* < 0.05) were observed between the Qinghai Han and the following groups: the Malaysian at 13 loci, the Tibetan and She at 11 loci, the Uygur and Shui at 10 loci, the Zhuang at five loci, the Dong and Yi at three loci and the Hui, Guangdong Han and Russian at two loci. No significant differences were observed among the Qinghai Han, Jiangsu Han, and Shaanxi Han groups. The highest ethnic diversity was observed at the *D18S51* locus, where significant differences were found in eight out of 13 compared groups. The lowest ethnic diversity was observed at *CSF1PO* and *TPOX* loci, where significant differences were found in only three out of 13 compared groups.

**Table 3.** The *P-*values of pairwise comparisons between Chinese Qinghai Han population and other groups at 13 overlapping STR loci based on the method of AMOVA.

Populations	*CSF1PO*	*D13S317*	*D16S539*	*D18S51*	*D21S11*	*D3S1358*	*D5S818*	*D7S820*	*D8S1179*	*FGA*	*TH01*	*TPOX*	*vWA*
Hui	0.786 9	0.891 5	0.995 1	0.008 8	0.663 7	0.988 3	0.164 2	1.000 0	0.508 3	1.000 0	0.291 3	0.081 1	0.028 4
She	0.000 0	0.000 0	0.001 0	0.012 7	0.000 0	0.481 9	0.003 9	0.000 0	0.000 0	0.001 0	0.002 0	0.307 9	0.000 0
Shui	0.843 6	0.001 0	0.002 0	0.000 0	0.000 0	0.049 9	0.052 8	0.045 9	0.000 0	0.000 0	0.006 8	0.001 0	0.075 3
Dong	0.275 7	0.021 5	0.060 6	0.255 1	1.000 0	0.000 0	0.754 6	0.702 8	0.000 0	0.142 7	0.334 3	0.785 9	0.560 1
Uygur	0.000 0	0.000 0	0.004 9	0.000 0	0.000 0	0.002 0	0.000 0	0.002 9	0.404 7	0.223 9	0.000 0	0.421 3	0.000 0
Yi	0.656 9	0.881 7	0.081 1	0.149 6	0.000 0	0.000 0	0.050 8	0.380 3	0.112 4	0.017 6	0.106 6	1.000 0	1.000 0
Zhuang	0.819 2	0.763 4	0.581 6	0.000 0	1.000 0	0.021 5	0.937 4	0.027 4	0.000 0	0.199 4	0.011 7	1.000 0	0.098 7
Guangdong Han	0.960 9	1.000 0	1.000 0	0.134 9	1.000 0	0.313 8	0.870 0	0.339 2	0.004 9	0.084 1	0.027 4	0.098 7	0.373 4
Jiangsu Han	0.927 7	0.793 7	0.128 1	0.181 8	0.698 9	1.000 0	1.000 0	1.000 0	0.764 4	0.910 1	0.993 2	1.000 0	1.000 0
Malaysian	0.034 2	0.000 0	0.000 0	0.000 0	0.001 0	0.010 8	0.000 0	0.000 0	0.000 0	0.000 0	0.000 0	0.000 0	0.000 0
Russian	0.185 7	0.003 9	0.253 2	0.013 7	0.320 6	0.277 6	1.000 0	0.928 6	0.271 8	0.339 2	1.000 0	0.472 1	0.296 2
Shaanxi Han	0.779 1	0.303 0	0.833 8	0.696 0	0.999 0	0.154 5	0.772 2	0.962 9	1.000 0	1.000 0	0.893 5	0.336 3	0.371 5
Tibetan	1.000 0	0.000 0	0.000 0	0.000 0	0.004 9	0.029 3	0.004 9	0.000 0	0.006 8	0.056 7	0.002 0	0.001 0	0.000 0

### Population structure clustering analysis of the 14 populations

**Figure 1.**
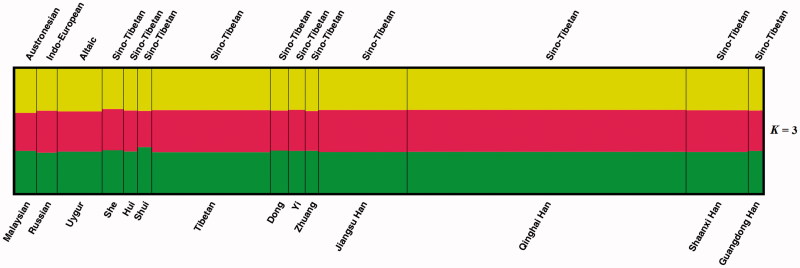
Cluster analysis by structure assuming *K* = 3. Population names are labeled beneath the plot and population languages above the plot.

We explored the population structures of the Qinghai Han and other published populations by the Structure program. Different *K* settings (*K* = 2–7) for the 13 overlapping loci dataset are shown in Supplementary Figure S1. Population components of the 14 populations at *K* = 3 are shown in Figure 1. Similar population component distributions were observed for all groups analysed, indicating a lack of population structure among these populations. Hence, the Qinghai Han group and other referenced populations showed no clear population structure in the present analysis.

### Genetic distance (D_A_ and *F*_ST_) analysis among the 14 populations

**Figure 2.**
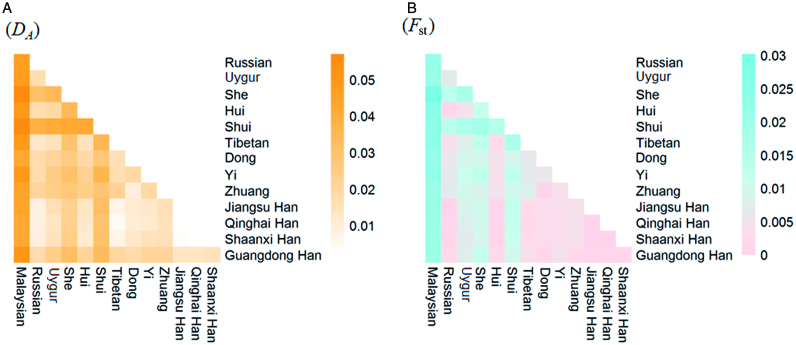
The genetic distance analysis among Qinghai Han and other reference populations. (A) Heatmaps of genetic distance (*D_A_*) and (B) fixation index (*F*_ST_) of the Qinghai Han population and other reference populations.

Pairwise genetic distances of the Qinghai Han population and other reference populations are shown in Figure 2 and Supplementary Tables S1 and S2. As shown in Figure 2(A), close relative genetic distances were observed between the Qinghai Han and Shaanxi Han and Jiangsu Han populations, while the largest distance was seen with the Malaysian (0.042 2). Similar results can be discerned from Figure 2(B), which indicates that the Qinghai Han have close relationships with Shaanxi Han and Jiangsu Han populations.

### PCA and MDS analysis of the Qinghai Han population and other reference populations

**Figure 3.**
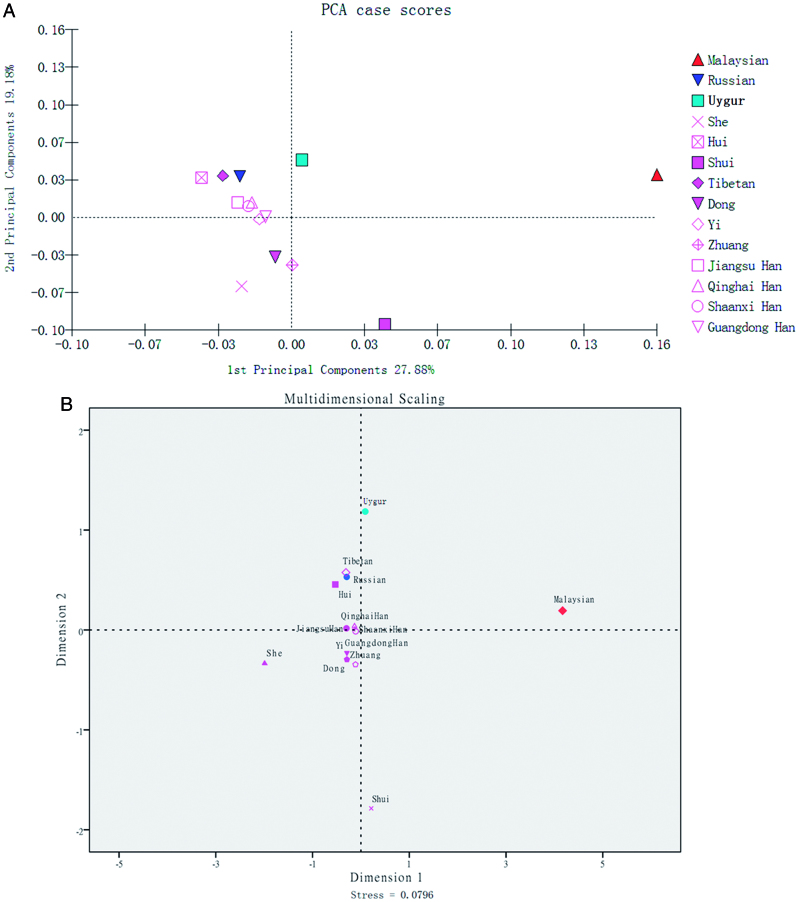
Principal components analysis (A) and multidimensional scaling analysis (B) of the Qinghai Han population and 13 other groups.

PCA of the Qinghai Han population and 13 other reference groups was performed according to the normalized allelic frequencies of the shared STR loci. As shown in Figure 3(A), the first two principal components contributed to 47.06% of the total variance, with the first and second components accounting for 27.88% and 19.18%, respectively. In Figure 3(A), the Qinghai Han group is clustered in the upper left quadrant, near the centre, close to the Shaanxi Han and Jiangsu Han groups. Similarly, most Han populations, including the Qinghai Han population, formed one cluster, which is located in the centre of the MDS plot. The proximity of these Han populations indicates their similar genetic components.

### Phylogenetic reconstructions of the Qinghai Han population and other Chinese populations

**Figure 4.**
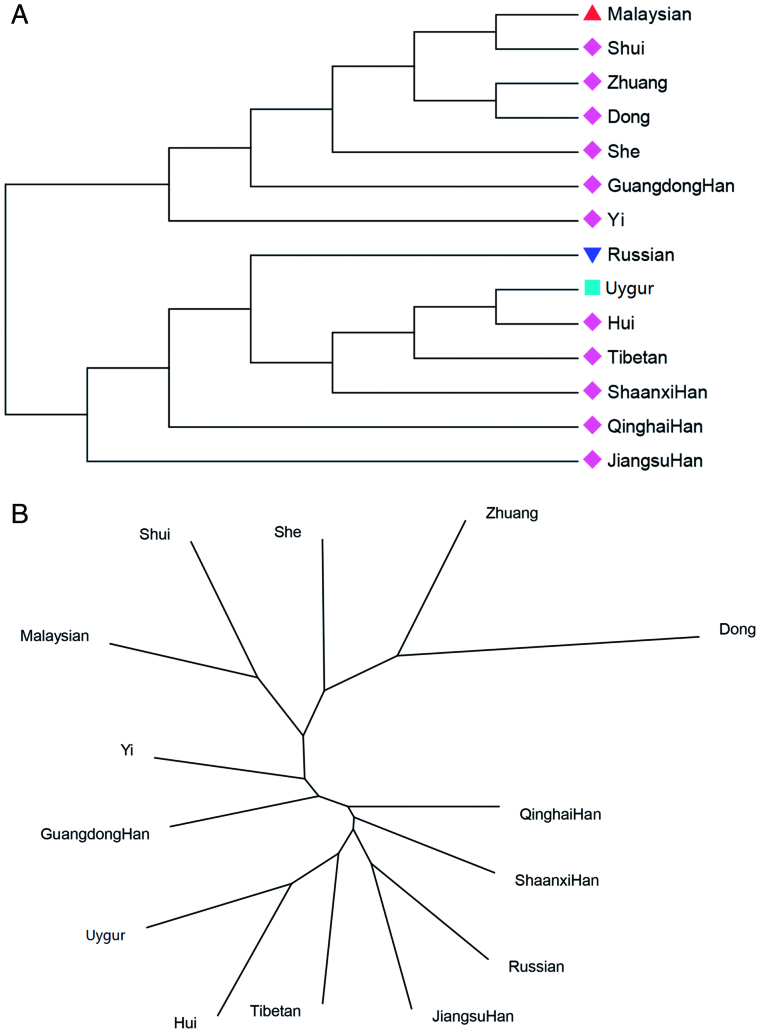
Phylogenic reconstructions of Qinghai Han population and other 13 populations based on allelic frequencies of the same STR loci. The Neighbor-Joining tree of these 14 populations was constructed using MEGA software (A); another phylogenetic tree was plotted using PHYLIP software (B).

Phylogenetic analysis was used to explore genetic relationships between the Qinghai Han group and other populations, as shown in Figure 4. The Neighbor-Joining tree method (Figure 4(A)) demonstrated that the Qinghai Han population formed a sub-branch of the tree with Shaanxi Han and Jiangsu Han populations. Another phylogenetic tree (Figure 4(B)) also showed that the Qinghai Han population was close to Shaanxi Han and Jiangsu Han populations. These phylogenetic results are consistent with the findings of the interpopulation differentiation study, genetic distance analysis, PCA and MDS described above, which likely reflects similar genetic distributions of Qinghai Han, Shaanxi Han and Jiangsu Han populations.

## Conclusion

The present study indicated that the loci examined were highly polymorphic in the studied Qinghai Han population. We also found that the Qinghai Han population has the close genetic relationships with Shaanxi Han and Jiangsu Han populations. These results suggest that the loci can be used for paternity testing and forensic human identification, and could also provide information about the genetic relationships between Qinghai Han and other groups.

## Compliance with ethical standards

The experimental protocol in the present research involving human blood samples conformed to the human and ethical research principles of Xi’an Jiaotong University Health Science Center.

## Supplementary Material

Supplemental Material
