# Supplementary material for: Genetic and structural characterization of 20 autosomal short tandem repeats in the Chinese Qinghai Han population and its genetic relationships and interpopulation differentiations with other reference populations
Source: Forensic Sci Res. 2018 Jul 18;3(2):145–52. doi: 10.1080/20961790.2018.1485199 (PMC6197092; doi:10.1080/20961790.2018.1485199)
Supplement: Supplemental Material [file TFSR_A_1485199_SM4239.zip › Supplementary Table 1.docx]

| Populations | Malaysian | Russian | Uygur | She | Hui | Shui | Tibetan | Dong | Yi | Zhuang | Jiangsu Han | Qinghai Han | Shaanxi Han |
| --- | --- | --- | --- | --- | --- | --- | --- | --- | --- | --- | --- | --- | --- |
| Russian | 0.0468 |  |  |  |  |  |  |  |  |  |  |  |  |
| Uygur | 0.0474 | 0.0166 |  |  |  |  |  |  |  |  |  |  |  |
| She | 0.0570 | 0.0309 | 0.0343 |  |  |  |  |  |  |  |  |  |  |
| Hui | 0.0485 | 0.0170 | 0.0189 | 0.0347 |  |  |  |  |  |  |  |  |  |
| Shui | 0.0568 | 0.0388 | 0.0410 | 0.0427 | 0.0421 |  |  |  |  |  |  |  |  |
| Tibetan | 0.0487 | 0.0130 | 0.0151 | 0.0299 | 0.0149 | 0.0356 |  |  |  |  |  |  |  |
| Dong | 0.0421 | 0.0180 | 0.0213 | 0.0262 | 0.0196 | 0.0276 | 0.0159 |  |  |  |  |  |  |
| Yi | 0.0491 | 0.0183 | 0.0254 | 0.0317 | 0.0224 | 0.0364 | 0.0171 | 0.0196 |  |  |  |  |  |
| Zhuang | 0.0441 | 0.0218 | 0.0248 | 0.0278 | 0.0245 | 0.0295 | 0.0224 | 0.0090 | 0.0194 |  |  |  |  |
| Jiangsu Han | 0.0449 | 0.0078 | 0.0156 | 0.0231 | 0.0133 | 0.0318 | 0.0074 | 0.0116 | 0.0130 | 0.0156 |  |  |  |
| Qinghai Han | 0.0422 | 0.0076 | 0.0133 | 0.0235 | 0.0125 | 0.0301 | 0.0053 | 0.0107 | 0.0118 | 0.0150 | 0.0020 |  |  |
| Shaanxi Han | 0.0427 | 0.0087 | 0.0144 | 0.0244 | 0.0118 | 0.0308 | 0.0068 | 0.0122 | 0.0146 | 0.0159 | 0.0040 | 0.0028 |  |
| Guangdong Han | 0.0507 | 0.0177 | 0.0249 | 0.0328 | 0.0214 | 0.0335 | 0.0198 | 0.0185 | 0.0214 | 0.0209 | 0.0139 | 0.0136 | 0.0149 |

Supplementary Table 1: The genetic distances (*D_A_*) of Qinghai Han population and other compared populations based on 13 overlapping STRs.
