# Supplementary material for: Genetic and structural characterization of 20 autosomal short tandem repeats in the Chinese Qinghai Han population and its genetic relationships and interpopulation differentiations with other reference populations
Source: Forensic Sci Res. 2018 Jul 18;3(2):145–52. doi: 10.1080/20961790.2018.1485199 (PMC6197092; doi:10.1080/20961790.2018.1485199)
Supplement: Supplemental Material [file TFSR_A_1485199_SM4239.zip › Supplementary Table 2.docx]

Supplementary Table 2: The fixation index (*F*_ST_) of Qinghai Han population and other published populations based on 13 same STR loci.

| Populations | Malaysian | Russian | Uygur | She | Hui | Shui | Tibetan | Dong | Yi | Zhuang | Jiangsu Han | Qinghai Han | Shaanxi Han |
| --- | --- | --- | --- | --- | --- | --- | --- | --- | --- | --- | --- | --- | --- |
| Russian | 0.0226 |  |  |  |  |  |  |  |  |  |  |  |  |
| Uygur | 0.0212 | 0.0068 |  |  |  |  |  |  |  |  |  |  |  |
| She | 0.0302 | 0.0138 | 0.0176 |  |  |  |  |  |  |  |  |  |  |
| Hui | 0.0253 | 0.0014 | 0.0045 | 0.0120 |  |  |  |  |  |  |  |  |  |
| Shui | 0.0247 | 0.0140 | 0.0173 | 0.0191 | 0.0149 |  |  |  |  |  |  |  |  |
| Tibetan | 0.0245 | 0.0037 | 0.0077 | 0.0134 | 0.0025 | 0.0171 |  |  |  |  |  |  |  |
| Dong | 0.0219 | 0.0055 | 0.0091 | 0.0095 | 0.0044 | 0.0091 | 0.0062 |  |  |  |  |  |  |
| Yi | 0.0230 | 0.0062 | 0.0112 | 0.0127 | 0.0039 | 0.0136 | 0.0062 | 0.0060 |  |  |  |  |  |
| Zhuang | 0.0209 | 0.0072 | 0.0097 | 0.0089 | 0.0049 | 0.0100 | 0.0082 | 0.0000 | 0.0040 |  |  |  |  |
| Jiangsu Han | 0.0219 | 0.0006 | 0.0087 | 0.0102 | 0.0011 | 0.0125 | 0.0027 | 0.0035 | 0.0040 | 0.0046 |  |  |  |
| Qinghai Han | 0.0204 | 0.0013 | 0.0077 | 0.0098 | 0.0013 | 0.0121 | 0.0023 | 0.0033 | 0.0035 | 0.0043 | 0.0000 |  |  |
| Shaanxi Han | 0.0208 | 0.0011 | 0.0070 | 0.0091 | 0.0008 | 0.0111 | 0.0025 | 0.0031 | 0.0042 | 0.0041 | 0.0000 | 0.0000 |  |
| Guangdong Han | 0.0209 | 0.0019 | 0.0077 | 0.0122 | 0.0021 | 0.0101 | 0.0041 | 0.0018 | 0.0047 | 0.0019 | 0.0015 | 0.0017 | 0.0018 |
