# Supplementary figures and images for: Genetic and structural characterization of 20 autosomal short tandem repeats in the Chinese Qinghai Han population and its genetic relationships and interpopulation differentiations with other reference populations
Source: Forensic Sci Res. 2018 Jul 18;3(2):145–52. doi: 10.1080/20961790.2018.1485199 (PMC6197092; doi:10.1080/20961790.2018.1485199)

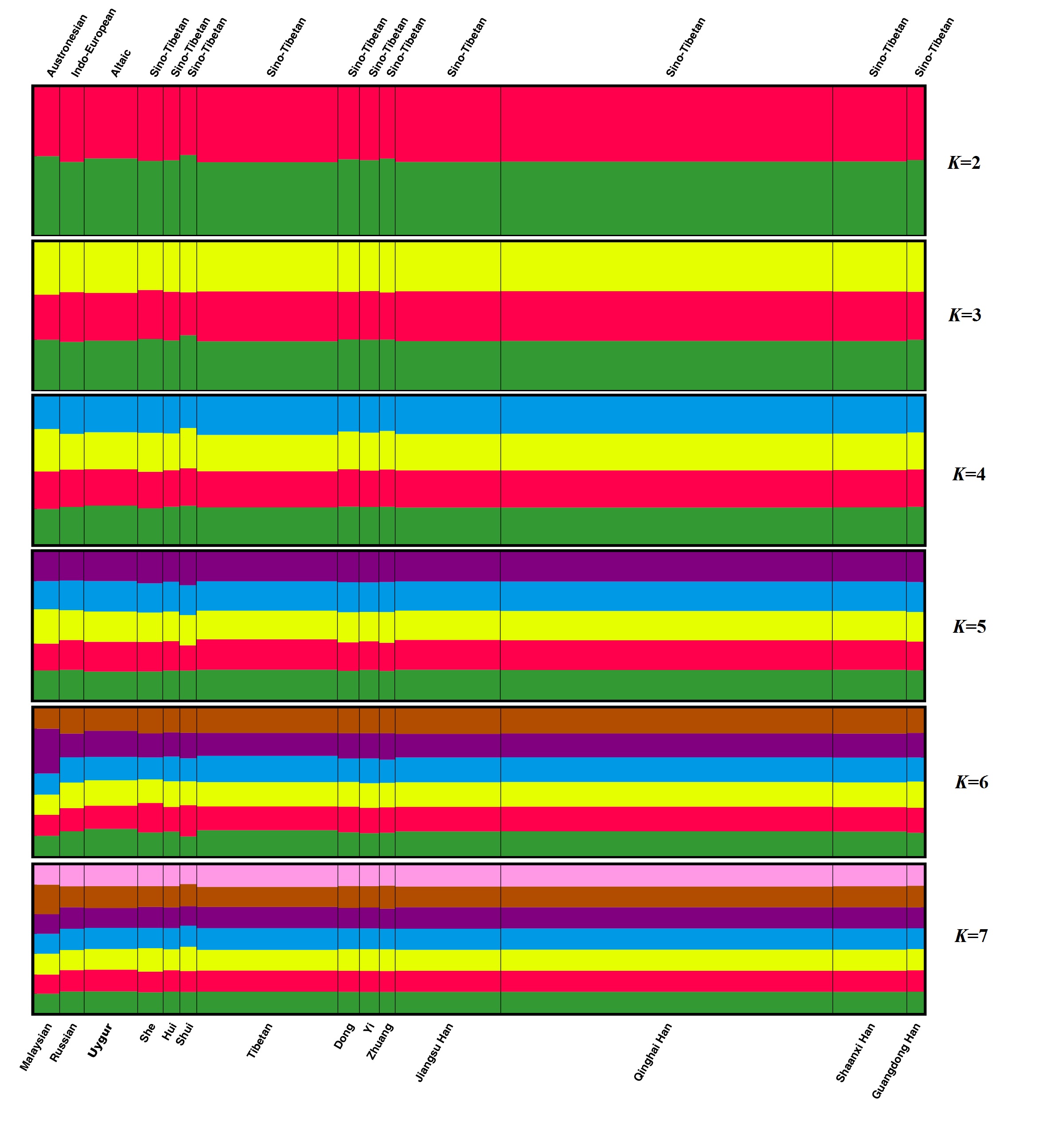

Supplement: Supplemental Material [file TFSR_A_1485199_SM4239.zip › Supplementary Figure 1.jpg]
